# Brainstem serotonin amplifies nociceptive transmission in a mouse model of Parkinson’s disease

**DOI:** 10.1038/s41531-024-00857-1

**Published:** 2025-01-07

**Authors:** Zoé Grivet, Franck Aby, Aude Verboven, Rabia Bouali-Benazzouz, Benjamin Sueur, François Maingret, Frédéric Naudet, Thibault Dhellemmes, Philippe De Deurwaerdere, Abdelhamid Benazzouz, Pascal Fossat

**Affiliations:** 1 Université de Bordeaux, Institut des Maladies Neurodégénératives, Bordeaux, France; 2 CNRS, Institut des Maladies Neurodégénératives, Bordeaux, France; 3 Université de Bordeaux, Institut des neurosciences cognitives et intégratives d’aquitaine, Bordeaux, France; 4 CNRS, Institut des neurosciences cognitives et intégratives d’aquitaine, Bordeaux, France

**Keywords:** Parkinson's disease, Diseases of the nervous system

## Abstract

Parkinson’s disease arises from the degeneration of dopaminergic neurons in the substantia nigra pars compacta, leading to motor symptoms such as akinesia, rigidity, and tremor at rest. The non-motor component of Parkinson’s disease includes increased neuropathic pain, the prevalence of which is 4 to 5 times higher than the general rate. By studying a mouse model of Parkinson’s disease induced by 6-hydroxydopamine, we assessed the impact of dopamine depletion on pain modulation. Mice exhibited mechanical hypersensitivity associated with hyperexcitability of neurons in the dorsal horn of the spinal cord (DHSC). Serotonin (5-HT) levels increased in the spinal cord, correlating with reduced tyrosine hydroxylase (TH) immunoreactivity in the nucleus raphe magnus (NRM) and increased excitability of 5-HT neurons. Selective optogenetic inhibition of 5-HT neurons attenuated mechanical hypersensitivity and reduced DHSC hyperexcitability. In addition, the blockade of 5-HT_2A_ and 5-HT_3_ receptors reduced mechanical hypersensitivity. These results reveal, for the first time, that PD-like dopamine depletion triggers spinal-mediated mechanical hypersensitivity, associated with serotonergic hyperactivity in the NRM, opening up new therapeutic avenues for Parkinson’s disease-associated pain targeting the serotonergic systems.

## Introduction

Parkinson’s disease (PD) is the second most common neurodegenerative disease affecting 2–3% of the population over the age of 60–65, an estimated 0.3% of the general population^1^. It is identified as the fastest growing neurological disorder in terms of deaths and disability^2^. PD is also characterized by non-motor symptoms that appear even before motor deficits, and worsen as the disease progresses^3^. Among these non-motor symptoms, pathological pain is one of the more prominent and debilitating affecting up to 95% of patients with PD who qualified it as one of the most troublesome non-motor symptoms throughout the disease^4,5^. Moreover, PD patients show a decrease in pain threshold, but not in tactile threshold, suggesting specific impairment of nociceptive transmission^6^. Pain in PD is a consequence of the progressive neurodegeneration notably altering dopamine (DA) neurons of the substantia nigra pars compacta (SNc). Therefore, nociceptive pain, such as musculo-skeletal symptoms, is improved by DA replacement drugs such as Levodopa (L-DOPA)^7^. However, up to 35% of PD patients have a refractory neuropathic pain component not directly associated with DA depletion and treated with antidepressant^8^. Consequently, the loss of DA neurons may partly explain the changes in pain thresholds but the absence of a total recovery of these symptoms with dopaminergic medication suggests also a role of non-dopaminergic mechanisms in the appearance or maintenance of pain symptoms in PD^3,9^. Finally, clinical studies failed to demonstrate peripheral nerve alteration in Parkinson’s disease patients suggesting a central origin of pain hypersensitivity^4^. Recent findings in PD revealed that DA depletion is also accompanied by altered activity of the serotonergic (5-HT) system^10,11^. Human’s studies showed a decrease of 5-HT levels in specific areas of the brain (Striatum, SNc, several cortical areas, thalamus and hypothalamus)^10^ and described neuronal degradation and presence of Lewy bodies in the dorsal and median raphe nuclei^12,13^. In addition, some showed a decrease of free and total 5-HT and its related substances in the cerebrospinal fluids from patients with PD^14,15^. Although these markers of 5-HT dysfunction are correlated with motor dysfunctions^10,15^ or neuropsychiatric symptoms as depression^16,17^ yet no correlation has been shown with pain. Still, post-mortem studies have shown a change in the integrity of the raphe nuclei, which contain the majority of the brain’s 5-HT neurons, or a change in the level of 5-HT or 5-hydroxy-indol-acetic-acid (5HIAA) in different areas of the brain^10^. Biochemical and neuroimaging studies have shown a reduction in the level of 5-HT in the cerebrospinal fluid of patients with PD, but not in the level of 5-HIAA^10,15^. Overall, the current hypothesis proposes that the reduced 5-HT system in PD is involved in both motor and non-motor symptoms of the disease.

Several animal models of PD have been developed, reproducing some of the motor and non-motor symptoms of the disease. Among the various models, intracerebral injection of the toxin 6-hydroxydopamine (6-OHDA) has been shown to induce robust loss of DA neurons, accompanied by severe motor impairments and pain hypersensitivity^18^. In this model, hypersensitivity to pain is associated with hyperexcitability of neurons in the dorsal horn of the spinal cord (DHSC) and an increased response to nociceptive stimulation, mimicking the situation observed in patients with an advanced stage of the disease^19^. However, the origin and underlying mechanisms of this hyperexcitability are still unknown. The basal ganglia (BG) are connected to the nociceptive networks. Therefore, the loss of DA neurons in the SNc in PD could alter the nociceptive network that projects to the DHSC and controls nociceptive transmission^20,21^. In particular, brainstem descending pathways, including 5-HT nuclei project to the DHSC^21–23^. The nucleus raphe magnus (NRM) of the brainstem is one of the main 5-HT nuclei projecting to the DHSC and is known to control nociceptive integration in the DHSC^23^. This nucleus has not been extensively studied compared to the dorsal raphe nucleus and a few studies reported conflicting results in patients with PD^24,25^. In the 6-OHDA model of PD, some rat studies suggested a decrease in 5-HT activity in the brainstem^26–28^. However, manipulation of the 5-HT system yelded conflicting results on the role of 5-HT projections to the DHSC and pain hypersensitivity^28^. Here, we propose to use an optogenetic approach to assess a direct link between NRM 5-HT neurons, the nociceptive response, and spinal hyperexcitability in PD. As we have previously observed a decrease in pain sensitivity by 5-HT activation in naive mice^29^, we postulate that 5-HT neurons in the NRM will exert an inhibitory tone on nociceptive transmission and decrease pain hypersensitivity in the context of PD. To test this hypothesis, we used a mouse model of the disease obtained by unilateral injection of 6-OHDA in the middle forebrain bundle (MFB). We first assessed for the alteration of the nociceptive transmission using behavioral and electrophysiological approaches, then we evaluated the alteration of the 5-HT network projecting to the spinal cord using high-performance liquid chromatography (HPLC), immunohistochemistry and ex vivo patch-clamp electrophysiology. Finally, we challenged 5-HT network using optogenetic and pharmacological manipulations. We confirmed the spinal hypersensitivity already observed in 6-OHDA rats and Parkinson’s disease patients, and unexpectedly showed that 5-HT neurons in the NRM are hyperactive and mediate descending facilitation promoting spinal hyperexcitability and pain hypersensitivity through 5-HT_2A_ and 5-HT_3_ receptors.

## Methods

### Animals

Adult (20 g–25 g) female and male wild-type (C57Bl6/J), and transgenic *ePet-cre* (B6.Cg-Tg (FeV-cre) 1Esd/J, Jackson Laboratory) mice were used in this study. *ePet-cre* mice were crossbreed with Ai9tdtomato line mice (Gt(Rosa)26Sortm6(CAG-td Tomato)Hze, Jackson lab, USA), in order to obtain *ePet-cre::Ai9tdtomato* mice. They were housed in the EOPS animal facility with free access to food and water, in constant temperature (21 ± 2 °C) and humidity levels (60%), under a 12:12 light/dark cycle, in a small social group setting (2–5 mice). All procedures were validated by the national and the local ethic committee of Bordeaux (CE50; Animal Facility PIV-EXPE, APAFIS#25605, APAFIS#32137 and APAFIS#26108; Animal Facilities of Neurocentre Magendie, APAFIS#21068). Animals were first submitted to a surgical procedure to inject 6-OHDA or saline. In the first set of experiments, a group of 26 shams and 29 6-OHDA mice was used. Three weeks after injection, animals were submitted to motor and sensory tests (see below). Then, either in vivo or patch-clamp electrophysiology was performed in the following days. Tissues were then extracted for immunohistochemistry. A group of 27 *ePet-cre* 6-OHDA mice was used for optogenetic (15 ArchT and 12 GFP).

### Surgery

Mice were anaesthetized in an induction chamber (isoflurane, 4%) then were placed into a stereotaxic frame (RWD, Shengzen, China) on a heating pad, head-fixed with ear bars and maintained at 1.5-2% isoflurane. Mice received ocular gel, betadine, and a subcutaneous injection of buprenorphine (100 µL, 0.1 mg/kg) and local injection of lidocaine (100 µL, 0.4 mg/kg).

6-OHDA or vehicle (NaCl 0.9%) was injected into the right MFB. 6-OHDA (Sigma, Saint-Quentin Fallavier, France) 12,5 mg in 2.5 ml of a solution of 0.09% NaCl containing 1% ascorbic acid used within 12 h. 1 µL of toxin was injected the MFB at the following coordinates (relative to bregma):AP = −1.2; ML = −1.3 and DV= from −4.85 to −4.65. An intraperitoneal injection of desipramine (Sigma-Aldrich, France) at 25 mg/kg was proceeded 30 min before the 6-OHDA injection. Post-surgery care was necessary during the first three weeks after the surgery.

In parallel, we proceed to stereotaxic injections of virus needed for the expression of the Archaerhodopsin (ArchT) or Green Fluorescent Protein (GFP) in the NRM. We injected a total of 600 nL (in 3 bolus of 200 nL, at coordinates from bregma AP/ML/DV −5,6/0/−5,6; −5,8/0/−5,6; −6,1/0/−5,7), of selected viruses: AAV-CAG-Flex-ArchT-YFP (4.2 × 10^12^ p/mL, UNC Vector Core) or AAV-CAG-Flex-GFP (8 × 10^12^ p/ml, UNC Vector Core).

Optical cannulae (KFP2301LZXX LC Ceramic Ferule NO flange OD 1,25 mm–ID 230 micron–conc <20 micron from AMS Technologies, associated with Ø200 μm Core TECS-Clad Multimode Optical Fiber, 0.39 NA from Thorlabs) were implanted above the surface of the DHSC.

Spinal cord implantations followed the protocol described in ref. ^30^. In anesthetized mice (see above surgery). A 1- to 2-cm incision was made slightly caudal to the peak of the dorsal hump to expose the lumbar spinal region. The T12 L1 vertebra of interest was identified, and then a small incision was made between the tendons and the vertebral column on both sides. The vertebra was then secured using spinal adapter clamps, and tissues were removed from the surface of the bone. A small hole was drilled approximately 1 mm from midline, centrally on the rostro-caudal axis on the left side. Finally, we sutured and cleansed the skin surrounding the dental cement securing the cannula implantation.

### Behavior experiments

For all behavioral experiments, mice were tester in the morning between 8 h and 12 h am. The experimenter was blinded from the animal group or treatment.

Locomotor symptoms induced by 6-OHDA were evaluated using cylinder test adapted from^31^. First, mice were placed individually inside a glass cylinder (diameter 144 mm). Each session of 2 min was videotaped and analyzed using EthoVision^®^ software (XT, Noldus Information Technology, NL). The spontaneous rotation preference has been assessed by counting the number of clockwise (ipsilateral) and anti-clockwise (controlateral) rotations.

Second, we placed the mice in an openfield of 40 × 40 cm for 10 min. Using the tracking Ethovision software, we evaluated the distance traveled by the animals.

To assess the mechanical thresholds, we used a set of Von Frey filaments of different forces (UgoBasile, Italy). Prior testing, mice were habituated 30 min to the testing apparatus. Von Frey filaments of different forces were applied to the plantar surface of the hind-paws. The simplified up-down method (SUDO), in which only five trials are done in the hind-paws, have been used^32^. In case of paw withdrawal, we used the next lowest filament. The last filament used, indicates the threshold weighted by the value of the 4th test. We converted the paw withdrawal threshold (PWT) measured to force (*g*) with the following equation: PWT_force_ = 10^(x*F+B)^ with PWT being the paw withdrawal threshold in grams used in the figures, F the filament number, x and B for filament numbered 2–7, *x* = 0.240 and *B* = −2.00, and for the filament numbered 7–14, *x* = 0.182 and B = −1.47.

We connected the 577 nm laser to the optical fiber, positioned at the lumbar spinal cord level, with its power set at 10 mW/mm². Continuous stimulation of 3 min long were delivered.

### Pharmacological experiments

To test the different antagonists, we inject a bolus of 10 µL of solution containing the drug or only saline as control directly at the level of the spinal cord via an intrathecal injection with a 10 µL Hamilton syringe equipped with a 30 G needle. Drug are all dissolved in NaCl 0.9% solution.

The concentration of the drug and the time let between the injection and the behavioral measure can be found in the following table:

**Table:** 

Antagonist	Reference	Concentration	Quantity	Time after injection
MDL11939	Millipore (SML0115)	0.1 mM	0.3 µg	30 min
Granisetron	Tocris (2903)	4 mM	14 µg	1 h

Granisetron and MDL11239 have been injected separately to respect their time before behavioral measure.

### High-performance liquid chromatography

The ipsilateral and contralateral lumbar spinal cord were rapidly extracted and frozen in cold isopentane (−35 °C). Tubes containing the lumbar cord samples were placed on ice, and rapidly wiped and weighed. Samples were then homogenized in 0.1 N HClO4 (100 µL), sonicated and centrifuged at 13,000 rpm for 30 min at 4 °C. The supernatants were injected into the HPLC system using a manual injector (Rheodyne 7725i, C.I.L. Cluzeau, Sainte-Foy-La-Grande, France) equipped with a 20 µL loop into the HPLC column (Hypersyl C18, 150 × 4.6 mm, 5 µm, C.I.L. Cluzeau) preceded by a Brownlee-Newgard precolumn (RP-8, 15 × 32 mm, 7 µm, C.I.L. Cluzeau). The filtered (0.22 mm Millipore filter) mobile phase was composed of (in mM): 60 NaH2PO4, 0.1 Disodium EDTA, 2-Octane-Sulfonic acid in deionized water (18 MW cm2) containing 7% methanol. The mobile phase was delivered by an HPLC pump (LC-20-AD, Shimadzu, France) at 1.2 mL/min. Monoamine detection was obtained with a coulometric cell (5011 coulometric cell, ESA, Paris, France) coupled to a programmable detector (Coulochem II, ESA). The potential of the electrodes was set to −270 and +350 mV. Signals were acquired using a Ulyss interface and stored on a computer using dedicated software (Azur, Toulouse, France). Moreover, a standard solution (1 ng/10 µL) was administered before and after each daily series of sample analysis. Results are expressed in pg/mg of tissue.

### Electrophysiological experiments

The brain was rapidly extracted and immerged in cold sucrose artificial cerebral spinal fluid (aCSF) solution composed of (in mM): 2 KCl, 26 NaHCO_3_, 1.15 NaH_2_PO_4_, 6 MgCl_2_, 0.2 CaCl_2_, 10 glucose, 220 sucrose, saturated with 95% O_2_-5% CO_2_. The brainstem is then separated from the rest of the brain and placed in the vibratome (LeicaVT1100S) intercalated between two blocks of 4% agarose. Slices of 250 µm think are transferred into an aCSF solution (in mM): 124 NaCl, 2.5 KCl, 1.25 NaH_2_PO_4_, 2 MgCl_2_, 2.5 CaCl_2_, 10 glucose, 25 NaHCO_3_ at 37 °C for an hour then at room temperature.

Whole-cell patch-clamp recordings were made using borosilicate electrodes (4–5 MΩ) filled with Biocytin (1%) and the following intracellular solution (in mM): 135 Kgluconate, 3.8 NaCl, 1 MgCl2, 10 HEPES, 0.1 EGTA, 0.4 Na_2_-GTP, 2 Mg-ATP, pH 7.21, 291 mOsm. Data were recorded using a MultiClamp 700B amplifier. Series and input resistances were measured on-line during recordings. Cells were rejected if series resistance changed by >20% during recordings. Action potentials were evoked by current steps injection (20 pA, 1 s) from −100 to +120 pA. Miniature synaptic currents were recorded at −60 mV in the presence of tetrodotoxine (1 µM). All recordings were analyzed off-line using ClampFit (version 10.6).

Mice were first anaesthetized with urethane to maintain level of activity of monoaminergic system (20%) and placed on a stereotaxic frame (Unimécanique, Asnières, France). Segments L4–L5 of the spinal cord were exposed. For light stimulation, an optical fiber was lowered as close as possible from the nervous tissue allowing 577 nm light stimulation at 8 mW/mm². Recording electrodes filled with NaCl 4% were built with glass capillaries from thin-walled borosilicate glass capillaries (outside diameter 1.5 mm; World Precision Instruments, Sarasota, FL, USA) and pulled using a puller (p-97; Sutter Instrument, Novato, CA, USA). Recordings were performed with an amplifier (DAM80, WPI, USA) connected to a CED1401 interface and recorded using Spike2 program (v7, CED, UK). Electric stimulations were performed with bipolar subcutaneous electrodes connected to an isolation box (isoflex, AMPI, Isr). Nociceptive field potentials were recorded using low pass filtered. Wide dynamic range (WDR) nature of the isolated units was assessed with electrical stimulation in the center of its receptive field and neurons with a fast (between 10 and 80 ms) and slow (between 80 and 150 ms) component were recorded using high pass filter.

Single-unit recordings of identified WDR have been done in sham and 6-OHDA animals. First, we determined that minimal stimulation allowed a slow response of the WDR. Then, we measured the response of the neurons to paw stimulation at 0.5, 1, 2, 3, 4, and 5 mA with one stimulation every 15 s. We then set the intensity as 3 times the threshold for optogenetic stimulations (continuous light for 1 min) and stimulated the paws again every 15 s. Finally, windup was assessed with a windup protocol, i.e., 15 stimulation at 1 Hz still at an intensity corresponding to three times the threshold.

### Immunohistochemistry

Mice were kept deeply anaesthetized and then transcardially perfused with 50 mL of NaCl 0.9% Heparine and 50 ml of 4% PFA. Both brain and spinal cord were dissected and fixed overnight in 4% PFA, and then cryopreserved in 12.5% PBS sucrose. After being frozen in Tissue-Tek O.C.T, brain and spinal cord sections were cut transversely at 20 µm on a cryostat (Leica CM3050S) and kept in PBS 1× Azide 0.2%.

After 3 bath wash of PBS 1×, sections were put free-floating in a solution of 0.1 M PBS/BSA 1%/triton 0.3% to permeabilize and saturate the slices. Slices were then bathed overnight at 4 °C in solution composed of primary antibody (see table below)/PBS 1×/BSA 1%. After being washed in PBS 1×, the secondary antibody (see table below) was added in 0.1 M PBS and BSA 0.1%.

**Table:** 

Fluorescent Antibody	Origin Species	Dilution	Reference
*Primary antibody*			
Anti-PhosphoErk	Rabbit	1/1000	Biotechne NBP1-19924
Anti-GFP	Chicken	1/1000	Aves GFP-1010
Anti-TPH2	Rabbit	1/2000-1/5000	Biotechne NB300-109
Anti-DBH	Rabbit	1/500	Abcam
Anti-TH	Mouse	1/2000	Sigma-Aldrich
*Secondary antibody*			
Anti-Rabbit Alexa 488	Goat	1/500	Thermo Fisher A11008
Anti-chicken Alexa 488	Goat	1/500	Thermo Fisher A-11039
Anti-Rabbit Alexa 647	Donkey	1/500	Thermo Fisher A21447
Streptavidin Alexa 488 or Alexa568	Sheep	1/500	Invitrogen S11223 or S11226

For TH and DBH quantification, we used DAB immunostaining. Slices are rinsed and permeabilized with PBS 1×/BSA 2%/Triton 100×0,3% solution for 30 min. Slices are then incubated overnight at 4 °C with anti-TH rabbit (1:5000, Novus Biologicals, NB300-109) or anti-DBH rabbit (1/500, Abcam). Slices are incubated 30 min with a secondary antibody anti-rabbit VisUCyte (R&D systems, VC003-125) and then washed again. Finally, the reaction is revealed via the chromogen Diaminobenzidin (DAB) and slices are then mounted on gelatine slides.

We acquired the images of the striatum, SNc, and A11 sections with the microscope Panoramic scan II 3DHISTECH. The images were analyzed with ImageJ Fiji software (National Institutes of Health, New York, USA). Only mice with a lesion of >80% in the SNc and >90% in the striatum were included in the present study.

Gray level of the striatum TH staining is defined as the difference between the mean intensity of the pixels of the striatum (i.e., TH + staining) and the mean intensity of the pixels located in the cortex (i.e., background staining). The gray level of the DHSC/RVM is defined as the difference between the mean intensity of the pixels of the DHSC/RVM (i.e., TH + staining) and the mean intensity of the pixel located in the blank surrounding area.

The number of TH cells in the SNc was obtained using the optical fractionator^33^ unbiased stereological method (Leica DM6000B, Mercator Pro, ExploraNova, France). Immunolabelled cells were counted by a blinded investigator on every 6 sections for the SNc (Counting frames: 60 × 40 μm, Spacing: 120 × 180 μm, number of sections: 5). We counted the number of A11 TH+ neurons manually using ImageJ and its cell counting plugin.

Images of the NRM of 5-HTCre*Ai9tdtomato animals were realized with the confocal microscope (Leica TCS SP5). The image were taken with ×20 oil immersion objective. A stack of 20 µm is imaged for each zone. TPH2, tdtomato, or PhosphoERK positive cells were counted with the Cell Counting plugin of ImageJ software.

### Statistics

As the groups contain less than 30 elements, we used non-parametric tests. When 2 groups are compared, we used Wilcoxon test if the data are paired and Mann–Whitney test otherwise. To look at the effect of 2 factors on data, we used two-ways ANOVA test for parametric values or Kruskal Wallis for non-parametric values. Graphs and tests are done with GraphPad Prism software. Data are presented as Mean ± SEM. Tests are considered significant if *p* < 0.05. *p < 0.05; **p < 0.01; ***p < 0.001).

## Results

### 6-OHDA injection-induced DA neuron lesion associated with motor impairments, mechanical hypersensitivity, and spinal hyperexcitability

**Fig. 1:**
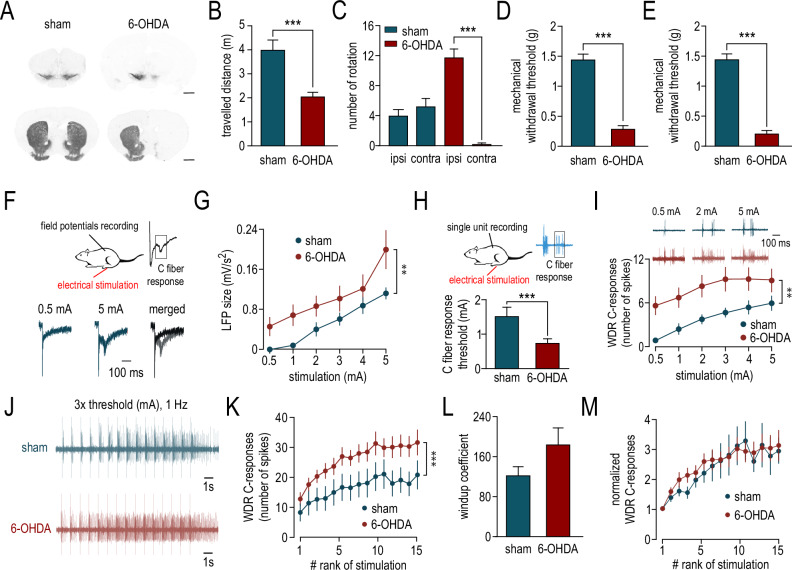
Mice model of hemiparkinsonism induces bilateral mechanical hypersensibility and spinal hyperexcitability. **A** Representative images of TH-(tyrosine hydroxylase) immunoreactive neurons in the substantia nigra pars compacta and the ventral tegmental area (upper panels) and TH- immunoreactive fibers in the striatum (lower panels) following stereotaxic injection of 6-OHDA (right panels) or vehicle (left panels) in the right medial forebrain bundle. Scale bar: 1 mm. **B** Summary data comparing the total distance traveled by sham (40.1 ± 33.9 m, *n* = 29) and 6-OHDA (20.7 ± 1.6 m, *n* = 26) mice in the openfield. **C** Summary data comparing the rotations ipsilateral or contralateral to the lesion in sham (3.9 ± 0.8 vs. 5.2 ± 0.1 respectively, *n* = 18) and 6-OHDA (11.7 ± 1.1 vs. 0.2 ± 0.1 respectively, *n* = 26) mice. **D** Summary data comparing the ipsilateral mechanical sensitivity of sham (1.46 ± 0.08 g, *n* = 29) and 6-OHDA (0.30 ± 0.05 g, *n* = 26) mice. **E** Summary data comparing the contralateral mechanical sensitivity of sham (1.44 ± 0.09 g, *n* = 29) and 6-OHDA (0.22 ± 0.03 g, *n* = 26) mice. **F** Cartoon (upper panel) and representative traces (lower panel) illustrating in vivo recordings of nociceptive field potentials in the DHSC (dorsal horn of the spinal cord). **G** Mean amplitude of the nociceptive field potentials plotted as a function of the intensity of stimulation in sham (*n* = 7) and 6-OHDA (*n* = 8) mice. **H** Upper panel: cartoon illustrating in vivo single unit recordings of DHSC WDR (wide dynamic range) neurons. Lower panel: summary data comparing the decrease in the C-fiber response threshold in 6-OHDA mice (sham: 1.53 ± 0.26 mA, *n* = 42 vs. 6-OHDA: 0.76 ± 0.12 mA, *n* = 47). **I** Mean amplitude of the WDR C-response plotted as a function of the intensity of stimulation in sham (*n* = 27) and 6-OHDA (*n* = 30) mice. The inset shows representative traces of WDR spikes in sham and 6-OHDA in response to 0.5, 2 and 5 mA of hind paw stimulation. **J** Representative traces of the short-term sensitization (windup) of WDR neurons in sham and 6-OHDA mice elicited by a train of 15 stimulations at 1 Hz at an intensity 3 times higher than the threshold. **K** Mean amplitude of the WDR C-response plotted as a function of the rank of stimulation in sham and 6-OHDA mice. **L** Summary data comparing the windup coefficient in sham (122.6 ± 19.7, *n* = 7) and 6-OHDA (160.3 ± 24.4, *n* = 20) mice. **M** Normalized mean amplitude of the WDR C-response plotted as a function of the rank of stimulation in sham and 6-OHDA mice.

We used a mouse model of hemiparkinsonism induced by unilateral 6-OHDA injection in the right MFB. This neurotoxin induced a subtotal lesion of DA neurons in the SNc and the ventral tegmental area (VTA) ipsilateral to the injection and a marked reduction in the ipsilateral DA fibers in the striatum compared to sham animals (Fig. 1A). Associated with this unilateral lesion, we observed a motor deficit, as shown by the decrease in the total distance traveled in the openfield (Fig. 1B, *p* < 0.001,), and by the almost exclusive ipsilateral rotations in the cylinder test in 6-OHDA mice (Fig. 1C, sham, *p* = 0.49, 6-OHDA, *p* < 0.001). We then evaluated the mechanical sensitivity of the animals using Von Frey filaments. We show that DA depletion dramatically decreased the mechanical withdrawal threshold in the ipsilateral (Fig. 1D, *p* < 0.0001) and contralateral (Fig. 1E, *p* < 0.0001) paws of the animals. In order to determine if the mechanical hypersensitivity is linked to spinal alteration, we recorded DHSC neurons that represent the first relay of nociceptive information. We first assessed the global nociceptive activity of the spinal cord by quantifying the local nociceptive field potentials (LFP) induced by noxious electrical peripheral stimulations (Fig. 1F). We show that LFP amplitude is increased in response to peripheral stimulations in 6-OHDA mice compared to sham (Fig. 1G, Sham vs 6-OHDA, *p* < 0.001, *F* = 21.28). We then recorded WDR neurons, which are located in the deep layers of the DHSC and represent a readout of the excitability of DHSC. WDR neurons responded to both innocuous (A-response) and noxious (C-response) peripheral stimulations (Fig. 1H). We focused here on the late nociceptive C-response following electrical stimulations applied to the plantar surface of the paw in the receptive field of WDR neurons. We measured the threshold for triggering a C-response of WDR neurons, and we show that this threshold is significantly decreased in 6-OHDA mice compared to sham animals (Fig. 1H, *p* < 0.001). The intensity-response curve for C-fibers is increased in 6-OHDA mice compared to sham animals (Fig. 1I, Sham vs 6-OHDA, *p* = 0.001, *F* = 34.03). These results indicate that 6-OHDA injection induced hyperexcitability of the WDR neurons in the DHSC. To investigate whether hyperexcitability in 6-OHDA mice is due to altered intrinsic properties of WDR neurons, we studied the windup, which is a form of short-term central sensitization elicited by repetitive stimulations at 3 times the threshold intensity for C-fibers and at low frequency (1 Hz) (Fig. 1J). We found that 8/25 and 20/47 WDR elicit windup in sham and 6-OHDA respectively (*p* = 0.64) and that in 6-OHDA mice the windup curve is higher than in sham (Fig. 1K, *p* < 0.001, *F* = 3.1). However, there is a slight but not significant modification of the windup coefficient suggesting that increased windup curve is not due to change of intrinsic properties (Fig. 1L, *p* = 0.4257). To confirm this possibility, we normalized the windup curve to the 1st response in order to focus on the amplification property. We show that amplification is strictly similar in 6-OHDA and sham mice suggesting that hyperexcitability in DHSC is due to alteration of the spinal network rather than intrinsic properties of WDR neurons (Fig. 1M, Sham vs 6-OHDA *p* = 0.34, *F* = 0.9).

### Decreased DA fibers in the RVM and increased electrical activity of 5-HT neurons

**Fig. 2:**
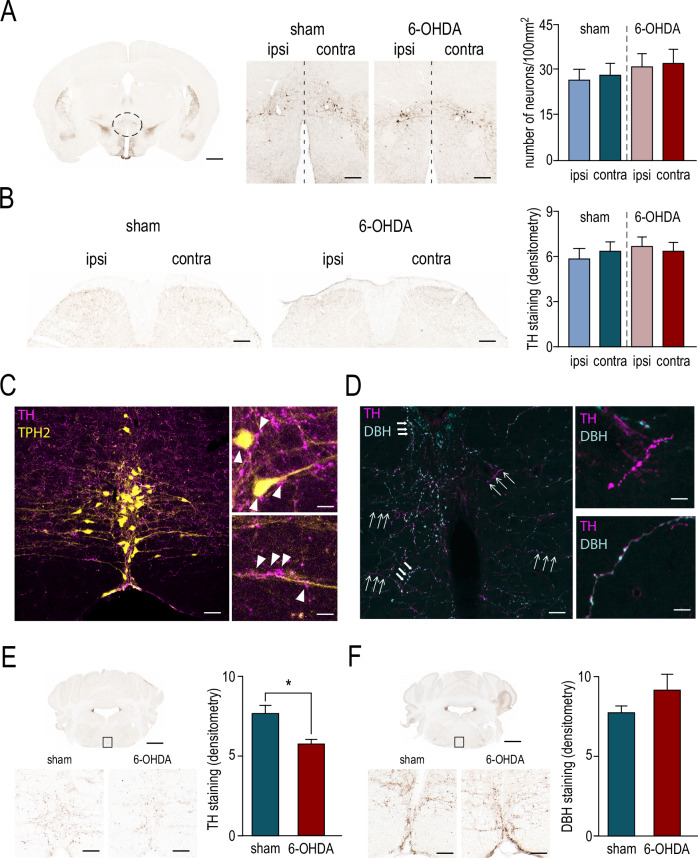
loss of dopaminergic fibers in the Nucleus Raphe Magnus but not in the spinal cord in the mice model of hemiparkinsonism. **A** Left: representative image of a DAB immunostaining against TH in the brain cross section containing the A11 hypothalamic nucleus. Scale bar: 1 mm. Middle: representative higher magnification images of A11 area showing TH- immunoreactive neurons in sham and 6-OHDA mice. Scale bar: 100 µm. Right: summary data comparing the quantification of TH- immunoreactive neurons in both ipsilateral and contralateral A11 area of sham (27.2 ± 3.2 and 28.8 ± 3.7, respectively, *n* = 16) and 6-OHDA (31.5 ± 4.3 and 32.8 ± 4.5, respectively, *n* = 23) mice. **B** Left: representative images of a DAB immunostaining against TH in the DHSC of sham and 6-OHDA mice. Scale bar: 100 µm. Right: summary data comparing the TH- immunoreactive fibers density in both ipsilateral and contralateral A11 area of sham (5.9 ± 0.6 and 6.5 ± 0.5 respectively, *n* = 12) and 6-OHDA (6.8 ± 0.5 and 6.4 ± 0.5 respectively, *n* = 18) mice. **C** Left: representative image of TH- (purple) and TPH2- (tryptophan hydroxylase 2, yellow) immunoreactive fibers in the NRM (nucleus raphe magnus). Scale bar: 100 µm. Right: higher magnification images illustrating the close apposition of TH- immunoreactive fibers to 5-HT (serotonin) neurons at both cell bodies (upper image) and fibers (lower image) levels. Scale bar: 20 µm. **D** Left: representative image of TH- (purple) and DBH- (dopamine beta-hydroxylase, cyan) immunoreactive fibers in the NRM. Scale bar: 100 µm. Right: higher magnification images showing fibers only TH-immunoreactive (thick arrowheads, upper image) and fibers both TH- and DBH- immunoreactive (thin arrowheads, lower image). Scale bar: 20 µm. **E** Upper left: representative image of a DAB immunostaining against TH in the NRM (square area). Scale bar: 120 µm. Lower left: higher magnification images of the NRM in sham and 6-OHDA mice. Scale bar: 50 µm. Right: summary data comparing the quantification of TH-immunoreactive fibers in the NRM of sham and 6-OHDA mice (7.7 ± 0.5, *n* = 20 and 5.8 ± 0.3, *n* = 20, respectively). **F** Upper left: representative image of a DAB immunostaining against DBH in the NRM (square area). Scale bar: 120 µm. Lower left: higher magnification images of the NRM in sham and 6-OHDA mice. Scale bar: 50 µm. Right: summary data comparing the quantification of DBH-immunoreactive fibers in the NRM of sham and 6-OHDA mice (7.9 ± 0.3, *n* = 20 and 9.3 ± 0.9, *n* = 20, respectively).

**Fig. 3:**
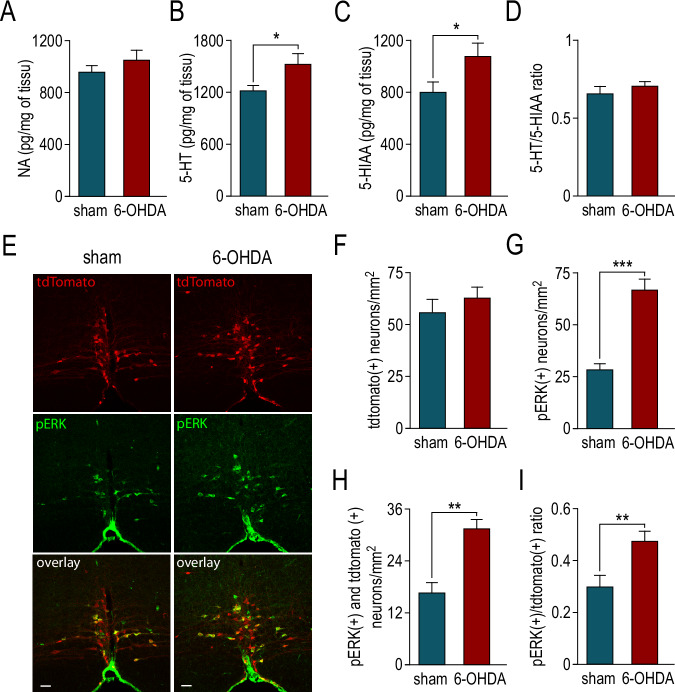
The activity of serotonergic neurons is increased in the mice model of hemiparkinsonism. **A** Summary data comparing the HPLC (high-performance liquid chromatography) dosage of NA (noradrenaline) from lumbar spinal cord of sham and 6-OHDA mice (962 ± 49 pg/mg, *n* = 6 and 1053 ± 75 pg/mg, *n* = 6, respectively). **B** Summary data comparing the HPLC dosage of 5-HT from lumbar spinal cord of sham and 6-OHDA mice (1222 ± 62 pg/mg, *n* = 6 and 1528 ± 123 pg/mg, *n* = 6, respectively). **C** Summary data comparing the HPLC dosage of 5-HIAA (5-hydroxyindolacetic acid) from lumbar spinal cord of sham and 6-OHDA mice (801 ± 82 pg/mg, *n* = 6 and 1079 ± 105 pg/mg, *n* = 6, respectively). **D** Summary data comparing the ratio between 5-HT and 5-HIAA in lumbar spinal cord of sham and 6-OHDA mice (0.65 ± 0.05, *n* = 6 and 0.71 ± 0.03, *n* = 6, respectively). **E** Representatives images of tdtomato (red) and pERK (phospho extracellular signal-regulated kinase) immunostaining (green) in the NRM of sham and and 6-OHDA mice. The overlapping clusters appear in yellow. Scale bar: 50 µm. **F** Summary data comparing tdtomato positive NRM neurons in sham and 6-OHDA mice (56.1 ± 6.5, *n* = 9 and 63.2 ± 5.5, *n* = 12, respectively). **G** Summary data comparing pERK positive NRM neurons in sham and 6-OHDA mice (28.6 ± 2.8, *n* = 10 and 67.1 ± 5.1, *n* = 13, respectively). **H** Summary data comparing both tdtomato and pERK positive NRM neurons in sham and 6-OHDA mice (16.7 ± 2.4, *n* = 10 and 31.6 ± 2.1, *n* = 13, respectively). **I** Summary data comparing the specific ratio of tdtomato positive NRM neurons also positive pERK in sham and 6-OHDA mice (0.3 ± 0.04, *n* = 10 and 0.47 ± 0.04, *n* = 13, respectively).

As DHSC neuronal activity is altered in our model, we focused on TH immunoreactivity in the DHSC of 6-OHDA mice and in the hypothalamic nucleus A11, considered as the DA nucleus projecting to the spinal cord^34^. TH immunoreactivity in the A11 and the DHSC remained unaffected (Fig. 2A, B, A11: number of TH+ neurons, *p* = 0.96: DHSC, Density of TH+ fibers, *p* = 0.7), suggesting that DA fibers projecting to the DHSC are preserved in our 6-OHDA mouse model. We, therefore, investigated descending pain pathways that project to the DHSC and modulate nociceptive transmission, in particular the NRM that contains 5-HT neurons^23,29,35^. We found that in NRM, 5-HT neurons are surrounded by dense TH-positive projections associated with close apposition in cell bodies and fibers (Fig. 2C). We found that these projections are both noradrenergic (NA) and dopaminergic (DA) (Fig. 2D). However, DA fibers are significantly reduced in the NRM of 6-OHDA mice (Fig. 2E, TH density, *p* < 0.05). NA fibers remained unaffected in part because NA neurons were protected by i.p. injection of desipramine in our model (see method) (Fig. 2F, DBH density; *p* = 0.18). We then assessed tissue levels of NA and 5-HT in the lumbar spinal cord of sham and 6-OHDA mice, using the HPLC coupled with electrochemical detection. Biochemical analysis showed that NA levels were unchanged in 6-OHDA animals compared with sham animals (Fig. 3A, test, *p* = 0.18). On the other hand, levels of 5-HT and its metabolite 5-HIAA were significantly increased (Fig. 3B, C, 5-HT: *p* < 0.05; 5-HIAA: *p* < 0.05). Interestingly, the 5-HT/5-HIAA ratio remained unchanged (Fig. 3D, *p* = 0.24). These results suggest that DA depletion is associated with an increased activity of 5-HT neurons.

**Fig. 4:**
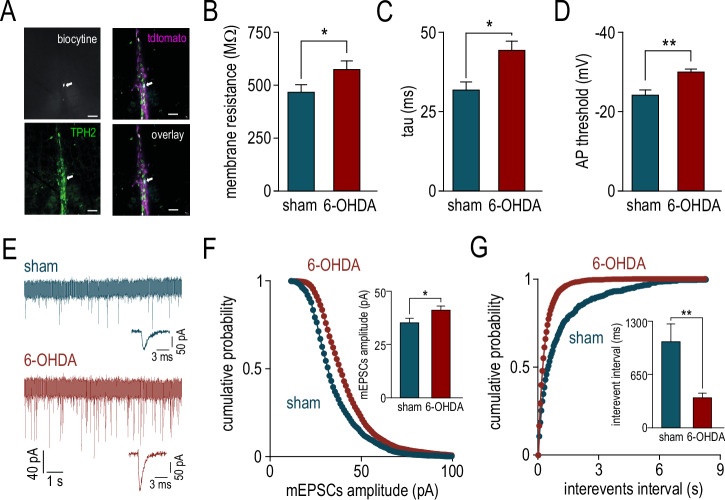
Both neuronal excitability and excitatory inputs onto 5-HT neurons of the NRM are increased in the mice model of hemiparkinsonism. **A** Representative images of a slice from the NRM illustrating a patched neuron filled by biocytine (top left) originally identified by tdtomato fluorescence (top right) and immunolabelled for TPH2 after patch-clamp experiment (bottom left). The overlapping neurons appear in white (bootm right). Scale bar: 50 µm. **B** summary data comparing the membrane resistance of NRM 5-HT neurons from sham and 6-OHDA mice (471 ± 37 MΩ, *n* = 12 and 579 ± 42 MΩ, *n* = 18, respectively). **C** summary data comparing the membrane capacitance of NRM 5-HT neurons from sham and 6-OHDA mice (tau value: 31 ± 2.4 ms, *n* = 13 and 43.4 ± 3.0 ms, *n* = 18, respectively). **D** summary data comparing the action potential threshold of NRM 5-HT neurons from sham and 6-OHDA mice (−25.7 ± 1.4 mV, *n* = 13 and −31.7 ± 0.8 mV, *n* = 18, respectively). **E** Representative traces showing mEPSCs recorded in NRM 5-HT neurons from sham and 6-OHDA mice in the presence of TTX (tetrodotoxin, 1 µM). The insets show expanded time scale single traces from both experiments. **F** Cumulative probability of mEPSCs amplitude of NRM 5-HT neurons from sham and 6-OHDA mice. The inset shows the pooled data from sham (35.5 ± 2.1 pA, *n* = 16) and 6-OHDA (41.5 ± 1.9 pA, *n* = 13) mice. **G** Cumulative probability of interevents intervals of mEPSCs of NRM 5-HT neurons from sham and 6-OHDA mice. The inset shows the pooled data from sham (1051 ± 212 ms, *n* = 16) and 6-OHDA (370 ± 56 pA, *n* = 13) mice.

To confirm this hypothesis, we investigated the expression of phosphorylated extracellular signal-regulated kinases (pERK), a marker of neuronal activity^36^, in 5-HT neurons of the NRM. Using *epet-cre::Ai9 td tomato* transgenic mice^29,37^, we compared the expression of pERK in NMR 5-HT neurons of 6-OHDA and sham animals (Fig. 3E). We first quantified the number of tomato positive neurons of the NRM (Fig. 3F) and found no difference between sham and 6-OHDA mice (*p* = 0.5). Then, using immunohistochemistry against pERK we show that the number of pERK+ neurons in the NRM significantly increased in 6-OHDA mice compared to sham animals (Fig. 3G, *p* < 0.001). This is due notably to an increased number of 5-HT neurons positive to pERK (Fig. 3H, *p* < 0.01). Finally, if we restrict quantification to NMR 5-HT neurons, we show that the proportion of 5-HT neurons positive to pERK in 6-OHDA mice is significantly higher than in sham animals (Fig. 3I, *p* < 0.01). We performed ex vivo recordings of 5-HT neurons, using the patch-clamp technique of NRM slices from both sham and 6-OHDA *epet-cre::Ai9 td tomato* mice (Fig. 4A). We investigated both the intrinsic properties and the synaptic connections of 5-HT neurons. We show that resting potential, action potential amplitude and shape of 5-HT neurons in the NRM are not modified in 6-OHDA mice compared to sham animals (resting potential; −57.3±1.2 mV sham, −61±1.5 mV 6-OHDA, *p* = 0.14; amplitude; 53±2.2 mV sham and 59.5±1.5 mV 6-OHDA, *p* = 0.40 and shape; 1.1±0.04 ms sham and 1.2±0.07 ms 6-OHDA, *p* = 0.40). However, we found a significant increase in the membrane resistance (Fig. 4B, *p* < 0.05), an increase in membrane time constant (Fig. 4C, *p* < 0.01), and a decrease of action potential threshold (Fig. 4D, *p* < 0.01). These results suggest that DA depletion increased the excitability of 5-HT neurons in line with their increased activity. Furthermore, we measured the miniature excitatory synaptic currents (mEPSCs) of 5-HT neurons (Fig. 4E). In 6-OHDA mice, we observed a significant increase in the amplitude of the mEPSCs (Fig. 4F, *p* < 0.05) as well as a significant decrease in inter-event intervals (Fig. 4G, *p* < 0.05) as compared to sham conditions. These results show an increased excitatory barrage onto 5-HT neurons that are also more excitable. Altogether, these results strongly suggest that 5-HT neurons of the NRM are hyperactive in the context of PD.

### Inhibition of 5-HT descending pathway in DA depleted animals reversed mechanical hypersensitivity

**Fig. 5:**
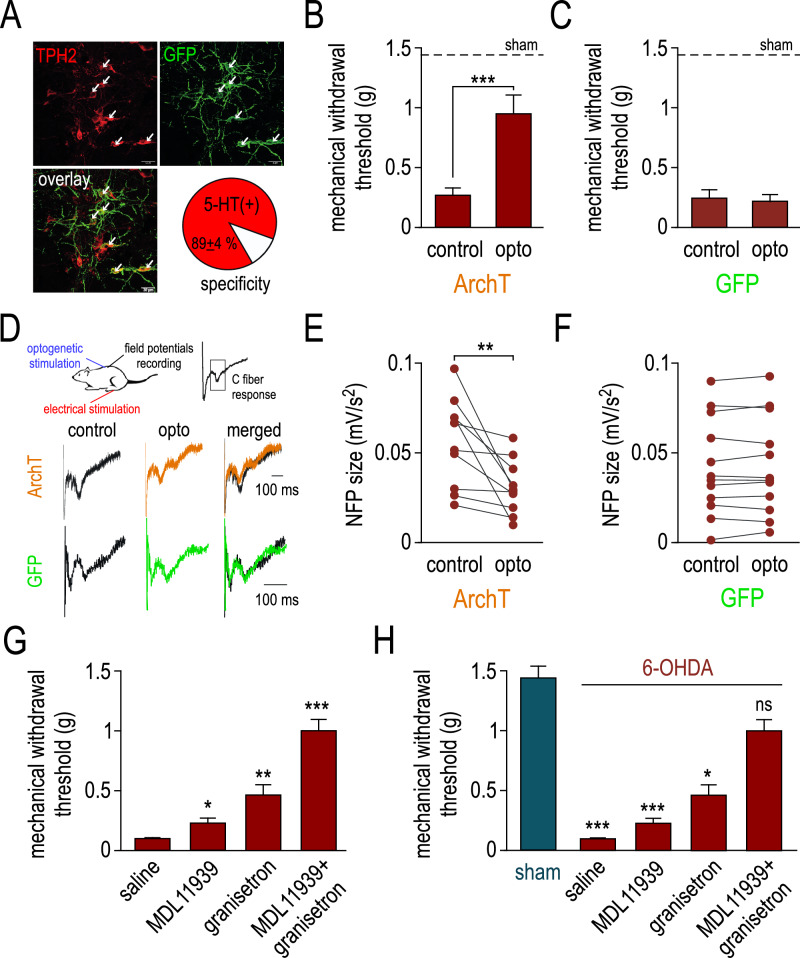
Inhibition of NRM 5-HT neurons reverses mechanical hypersensitivity in the mice model of hemiparkinsonism. **A** Representative images of the expression of GFP (green, top left) and THP2 (red, top right) after the virus infection of the NRM. The overlapping clusters, indicated by the arrows, appear in yellow (bottom left) and show a high specificity of the virus expression in 5-HT neurons (bottom right). Scale bar: 30 µm. **B** Summary data comparing the effect of the optogenetic ArchT stimulation on the mechanical sensitivity of 6-OHDA mice (control: 0.27 ± 0.06 g vs. opto: 0.95 ± 0.16 g, *n* = 15). The dashed line indicates the mechanical sensitivity of sham mice. **C** Same protocol as in (**B**) except that the expression of ArchT is replaced by GFP. Control: 0.26 ± 0.07 g vs. opto: 0.23 ± 0.05 g, *n* = 12). **D** Example traces of nociceptive field recordings in the DHSC of 6-OHDA mice expressing either ArchT or GFP before and during optogenetic stimulation of the 5-HT descending. **E** Summary data comparing the effect of the ArchT optogenetic stimulation on the amplitude of the nociceptive field potentials in 6-OHDA mice (control: 0.056 ± 0.008 mV/s^2^ vs. opto: 0.031 ± 0.005 mV/s^2^, *n* = 10). **F** Summary data comparing the effect of the GFP optogenetic stimulation on the amplitude of the nociceptive field potentials in 6-OHDA mice (control: 0.042 ± 0.008 mV/s^2^ vs. opto: 0.043 ± 0.008 mV/s^2^, *n* = 12). **G** Summary data comparing the effect of the intrathecal injection of 5-HT receptors antagonists on the mechanical sensitivity of 6-OHDA mice (saline: 0.11 ± 0.01 g, *n* = 8; MDL11939: 0.24 ± 0.04 g, *n* = 8; granisetron: 0.47 ± 0.09 g, *n* = 8; MDL11939+gransitron: 1.01 ± 0.09 g, *n* = 8). **H** same experiments as in (**G**) comparing the mechanical sensitivity of 6-OHDA mice in the presence of 5-HT receptors antagonists to sham mice (1.44 ± 0.09 g, *n* = 29).

We next assessed the role of 5-HT descending pathway in mechanical pain hypersensitivity and spinal hyperexcitability in 6-OHDA mice. We injected a cre-dependent adenovirus (AAV-dio-ARchT-GFP) in NRM of *epet-cre* mice allowing the specific expression of ArchT, an inhibitory opsin acting as a proton-pump, in 5-HT neurons (Fig. 5A). We used a group of *epet-cre* mice infected with an AAV-dio-GFP as a control group. We implanted optical fibers necessary for opsin activation above dorsal surface of the spinal cord to specifically inhibit 5-HT descending fibers (see method and Aby et al.^29^). Optogenetic inhibition of the 5-HT descending pathways significantly increased mechanical withdrawal threshold of 6-OHDA mice expressing ArchT but not GFP alone (Fig. 5B, C, *p* < 0.001 for ArchT and *p* = 0.75 for GFP). We then investigated the effect of the optogenetic inhibition on spinal hyperexcitability (Fig. 5D) and we show that optogenetic inhibition of 5-HT descending pathway decreased the global size of nociceptive LFP of the DHSC (Fig. 5E, F, *p* < 0.01 for ArchT and *p* = 0.48 for GFP). These results show that inhibition of 5-HT descending pathway from the NRM is able to reverse dorsal horn hyperexcitability and mechanical hypersensitivity.

### 5-HT_2A_ and 5-HT_3_ receptors are involved in mechanical hypersensitivity in 6-OHDA mice

Finally, we addressed the question of the 5-HT receptors involved in such nociceptive hypersensitivity. We performed intrathecal injections of 5-HT receptor antagonists and evaluate mechanical sensitivity in 6-OHDA mice. The selective 5-HTR_2A_ antagonist MDL11939 partially reversed mechanical withdrawal threshold (Fig. 5G, *p* < 0.05). The 5-HTR_3_ antagonist granisetron also significantly increased the mechanical threshold (Fig. 5G, *p* < 0.001). Finally, we tested the effect of simultaneous blockade of 5-HT_2A_ and 5-HT_3_ receptors. The combined intrathecal injection of granisetron and MDL11239 significantly increased the mechanical threshold to a level close to that of sham animals, suggesting a synergistic effect of the two antagonists (Fig. 5G, *p* < 0.01). To confirm this hypothesis, we plotted the different combinations and compared the difference with the values of the sham animals (Fig. 5H). We found that the combination of granisetron and MDL11239 induced an increase in mechanical threshold that was not different from that of the sham condition (*p* = 0.99).

## Discussion

In the present study, we confirm that DA neuron lesion-induced mechanical hypersensitivity is associated with dorsal horn neuron hyperexcitability in the 6-OHDA mouse model of hemiparkinsonism. This hyperexcitability is associated with a loss of TH fibers and hyperactivity of 5-HT neurons in the NRM. We unexpectedly observed that 5-HT neurons from the NRM exert a descending facilitation on nociceptive transmission and selective optogenetic silencing of 5-HT neurons reversed the pathological mechanical hypersensitivity and the spinal hyperexcitability. Finally, we show that the pathological hypersensitivity was reversed by the synergistic blockade of 5-HT_2A_ and 5-HT_3_ receptors that should be considered in the future as potential therapeutic routes.

We used a model of hemiparkinsonism obtained by the unilateral injection of 6-OHDA in the MFB of adult mice, inducing a dramatic unilateral lesion of DA neurons in the SNc and fibers in the striatum^38,39^. This model is widely used to study the pathophysiology of PD, in particular to mimic the advanced stage of the disease. Although it does not show accumulation of misfolded proteins such as alpha-synuclein aggregates, for which the genetic model may be more appropriate, this 6-OHDA model has several advantages, including the reproduction of motor and some non-motor signs, as well as the absence of inter-individual variability. Genetic models often generate highly complex phenotypes far beyond the symptoms or whose results have not been reproduced^18,40^. For instance, these models are based on the mutation of genes involved in the familial form of PD, alpha-synuclein (*SNCA*), LRRK2 gene (*PARK8*), parkin gene (*PARK2)* leading to contradictory results with motor impairments without dopaminergic lesions or the opposite. Only one genetic model has been used to test pain sensitivity and show mechanical and thermal hypersensitivity^41^. This model is a specific mutant mouse with a mutation in a transcription factor specific of dopaminergic neurons (*Pitx3*). Although strong nigro-striatal DA depletion was observed, the mutation generated deleterious side-effects not associated with PD, such as microphtalmia. Finally, the 6-OHDA model is the most reproducible and usable animal model of DA loss associated with the motor and non-motor symptoms of PD. In this 6-OHDA model, we observed motor impairments as shown by unilateral rotations in the cylinder test and reduced locomotor activity in the openfield test. We also observed bilateral allodynia as the mechanical threshold in the hind paw decreased. This result is in line with previous studies carried out in mice and patients^38,42^. In rodents, recent studies have used a 6-OHDA model without the protection of noradrenaline neurons and observed mechanical allodynia in mice and rats^27,43^. We and others have focused on DA depletion and protected noradrenaline neurons with desipramine. We observed the same mechanical hypersensitivity in rats and mice^19,44^. These results suggest that DA depletion is the key event for generating mechanical hypersensitivity whereas noradrenaline depletion is not. The results are more contrasted for thermal sensation since thermal hypersensitivity is observed or not in rats with or without desipramine^21,28,43,44^. Finally, our results in mice confirm previous observations in rats and demonstrate the validity of pure dopaminergic depletion for inducing mechanical hypersensitivity and studying its consequences.

We also show that neither A11 DA neurons nor DA fibers in the spinal cord were damaged. This contradicts recent observations suggesting a loss of DA in the spinal cord with MPTP mouse model of PD^45^. However, this is consistent with a previous anatomical study showing the integrity of DA A11 neurons in the hypothalamus in PD patients and no change in DA levels in the spinal cord^14,46^. This suggests that DA depletion indirectly affects the spinal cord microcircuitry. We also showed that unilateral 6-OHDA lesion induced hyperexcitability of dorsal horn neurons (DHN). We assessed the activity of DHN that respond to stimulation of peripheral nociceptive and non-nociceptive fibers. These neurons are called WDR neurons and are considered to be one of the main outputs from the DHSC^47^. Changes in WDR excitability are a good indicator of DHSC sensitization, a phenomenon that occurs following injury to the peripheral or central nervous system^48^. We show that WDR neurons increased their responsiveness to nociceptive inputs, which was present across all stimulation intensities. We and others have previously observed increased excitability and altered intensity responses of WDR neurons in the DHSC of rats following unilateral injection of 6-OHDA into the MFB, suggesting that spinal excitability is a key feature of 6-OHDA-induced nociceptive hypersensitivity in mammals^21,49,50^.

These changes in DHN excitability may have several origins and may be the consequence of local changes. For example, following peripheral nerve lesion that induced neuropathic pain, a neuroimmune response mediated through microglia activation is responsible for loss of inhibition and hyperexcitability^51,52^ Recent studies suggest that such mechanisms may be responsible for spinal hyperexcitability in PD, as they showed increased cFos activation in the DHSC and increased expression of iba1, a specific marker of microglia activation^26,27^. Astrocytes are also involved in pain control in DHSC^53^, and are also activated in DHSC in the 6-OHDA model of PD^27,54^.

Beyond spinal plasticity, the plasticity of descending pathways may be responsible for spinal hyperexcitability. Recent studies have shown that BG can detect nociceptive stimuli and modulate spinal cord neuronal activity, and DA depletion altered this interaction^21,22^. Midbrain and brainstem nuclei may also be involved, in particular the periaqueductal gray (PAG) and rostral ventromedial medulla (RVM). In the PD model, PAG neuronal activity is reduced with a loss of dopaminergic neurons and cfos activity and an increase in GABA immunoreactivity ipsilateral to the lesion^26,27^. GABA neurons in the PAG are known to participate in the descending facilitation that promotes pain hypersensitivity^55,56^ and project to the RVM.

In the RVM, three subtypes of neurons have been identified according to their response to nociceptive inputs: ON, OFF, and neutral cells^57,58^. ON cells are activated by nociceptive inputs while OFF cells are inhibited. ON cells mediate descending facilitation while OFF cells control descending inhibition. In contrast, neutral cells do not detect nociceptive inputs but their activation modulates nociceptive integration in the DHSC. This is the case for the 5-HT neurons of NRM^57^, which exert multiple actions on the spinal network^29,35,59^. ON, OFF and neutral cells are all involved in descending control of pain but belong to different neural circuits. When and how these different circuits are engaged is not yet fully understood, but the regular and tonic activity of monoaminergic neurons such as 5-HT neurons suggests that they exert a constant tone on spinal nociceptive neurons rather than acute activation^60^. Finally, opioids are one of the most potent analgesics acting through local spinal inhibition of nociceptive circuits or by inhibition of descending facilitation^57,58^. For example, opioids are known to activate OFF cells and inhibit ON cells^57^. Locally, in the spinal cord, enkephalin-expressing neurons exert an analgesic action and are inhibited by braistem GABA neurons^61^. Interestingly, the immunoreactivity of enkephaline and mu-opioid receptors is decreased in PD 6-OHDA model, which could also explain the hyperexcitability of the spinal cord^43^.

Finally, we found no modification of the windup, a form of short-term plasticity expressed by WDR suggesting that sensitization capabilities of 6-OHDA mice depend on spinal network rather than on WDR intrinsic properties. This result is different from what we observed in 6-OHDA rats where windup amplitude was increased in WDR neurons. However, this discrepancy may be explained by the different anesthetics used in the two studies. In rats, electrophysiological recordings were made under isoflurane anesthesia, whereas in mice we used urethane. This was justified by the fact that isoflurane inhibits the activity of 5-HT neurons^62,63^.

Motor impairments as well as nociceptive hypersensitivity and spinal cord neurons hyperexcitability are a consequence of the loss of dopaminergic nigro-striatal pathway. We investigated the involvement of the serotonergic system, in particular that of the descending pathway from the NRM to the spinal cord. The serotonergic system is known to be altered in PD patients^10,11^. For example, reduced levels of 5-HT and 5-HIAA have been found in many areas of the brain, mainly associated with degeneration of dorsal raphe neurons and motor disorders^10^. However, as almost all patients are treated with L-Dopa and L-Dopa can induce changes in serotonergic systems, the role of L-Dopa in reducing 5-HT is still debated. Finally, little is known about the degeneration of the 5-HT system projecting to the spinal cord including NRM. Yet this is an important source of 5-HT for the spinal cord and is known to control nociceptive transmission. A post-mortem study on the brains of PD patients showed the presence of protein aggregates in NRM, but without identifying the type of neurons, and another one showed a decrease in 5-HT levels in the spinal cord^14,24^. However, all the patients were treated with L-Dopa, which can interfere with 5-HT metabolism. In contrast, another study showed no degeneration of 5-HT neurons in the NRM of patients with PD^25^. In animal models, alterations in 5-HT appear to depend on the model used, protection of the NA system, and treatment with L-DOPA^26–28,64^.

In our context, we used the 6-OHDA model to reproduce the motor deficits and pain hypersensitivity induced by the midbrain dopaminergic depletion alone, which represents the main feature of PD. First, we show no difference in the number of 5-HT neurons in the NRM or in 5-HT fibers in the spinal cord. This is in contradiction with some studies suggesting a loss of 5-HT neurons or fibers in the DHSC^27,28^. However, in all these studies, NA neurons were not protected, unlike in our study, where we show dense connections between locus coeruleus (LC) and NRM. The loss of 5-HT neurons could be secondary to the loss NA neurons. On the other hand, we cannot exclude the fact that all these studies were carried out on rats whereas we used mice. Finally, the way in which 6-OHDA was applied is also different and could affect the 5-HT system differently. We also show increased levels of activity of 5-HT neurons in the NRM and 5-HT in the DHSC. With regard to the increased activity of 5-HT neurons, the results are in contradiction with a recent study suggesting a decrease in activity^26^. However, the authors used a cFos response to nociceptive stimuli, and we used the level of pERK. In the first case, the authors only showed that 5-HT neurons respond less to nociceptive stimuli, whereas we show a higher basal activity level of these neurons. Moreover, using patch-clamp recordings of 5-HT neurons identified in the NRM, we show that 5-HT neurons are more excitable and receive an increased barrage of mEPSCs in the 6-OHDA mice compared to sham animals. These results are comparable to those of a recent study showing increased excitability of dorsal raphe 5-HT neurons in the PD model^65^. This increased excitability is associated with morphological changes in 5-HT neurons. In the present study, our results also suggest morphological changes as we observed an increase in membrane resistance and time constant. We also observed a decrease in the action potential threshold that increased neuronal excitability. This decrease could be due to a change in the combination of voltage-gated sodium channels (Nav), as the different subunits have different activation thresholds^66^. Finally, we observed an increase in 5-HT levels in the DHSC while the dynamic of 5-HT degradation remained unchanged, and the 5-HT/5HIAA ratio was stable. These results suggest that the increase in 5-HT levels is not due to a deficiency in the pathway degradation but rather to an increase in the activity of 5-HT neurons. Altogether, our results demonstrate that severe damage to DA neurons alone induces hyperactivity of 5-HT system in the NRM.

We show that 5-HT neuron activity is increased and this increase is associated with spinal hyperexcitability and mechanical pain hypersensitivity. Manipulation of 5-HT system in animal model of PD show that serotonin-specific reuptake inhibitors (SSRIs) suppress mechanical allodynia in bilateral 6-OHDA model of PD and only in the contralateral paw in the hemiparkinsonism 6-OHDA model of PD^27,28^. By contrast, 5-HT neuron depletion in 6-OHDA model of PD with the toxin 5,7-DHT improves mechanical sensitivity suggesting that 5-HT neuron of the NRM mediate mechanical hypersensitivity^28^. In our study, we used an optogenetic approach to express ArchT opsin in NRM 5-HT-neurons. Activation of ArchT by green light is known to induce proton efflux, resulting in strong hyperpolarization^67^. To specifically target 5-HT fibers projecting to the spinal cord, we implanted an optical fiber above the DHSC to illuminate descending fibers^30^. Using this optogenetic approach, we show that inhibition of 5-HT fibers increased mechanical threshold and decreased the hyperexcitability of DHSC neurons. These results are in agreement with previous studies showing the involvement of 5-HT descending pathway in nociceptive transmission in different pain models^29,35,59^. This pro-nociceptive action of 5-HT neurons has already been shown in neuropathic pain following peripheral nerve lesion using the optogenetic manipulations^29,59^. In PD, as in peripheral neuropathy common mechanisms are present, such as microglia and astrocyte activation^43,51,68,69^. Pain hypersensitivity and pro-nociceptive role of 5-HT descending pathways could be induced by similar mechanisms such as disinhibition mechanisms^52^.

To directly associate spinal hyperexcitability and descending 5-HT pathways in our 6-OHDA mouse model of hemiparkinsonism, we used a pharmacological approach targeting 5-HT receptors. 5-HT is associated with 7 different receptors 5-HT1-7 and at least 15 subtypes^23^. A recent study identified at least 12 subtypes involved in DHSC responses to 5-HT receptor manipulation^70^. Our results show that at least two 5-HT receptors are involved in mechanical allodynia in the 6-OHDA model of PD. Facilitation of the descending 5-HT pathway has been observed in a variety of pathological contexts, including inflammation and neuropathic pain, and is thought to be associated with 5-HT_2A_ and 5-HT_3_ receptor subtypes^71,72^. Here we show that spinal hypersensitivity can be blocked by 5-HT_2A_ and 5-HT_3_ receptor antagonists. The involvement of the 5-HT_3_ receptor in pain in PD has been highlighted recently, as the 5-HT_3_ antagonist reversed mechanical hypersensitivity and decreased hyperexcitability of dorsal horn neurons^50^. In addition, another study suggested that 5-HT modulates TRPV1 nociceptor activity via 5-HT_3_ receptor in DHSC^73^. Our results confirm the pro-nociceptive role of 5-HT_3_ receptor, but the effect of the antagonist remains modest. We obtained complete recovery with the addition of 5-HT_2A_ antagonist. This result suggests a synergistic action of the blockade of 5-HT_2A_ and 5-HT_3_ blockade on mechanical allodynia. This synergistic effect could be due to the action of these two receptors in different neuronal subpopulations in the DHSC^74^. As the 5-HT_2A_ receptor is also a target for motor impairments in PD^75^, the combination of the two antagonists could be an interesting option to treat PD.

In conclusion, our results show that 6-OHDA-induced DA depletion in mice induced a spinally mediated pain hypersensitivity associated with an impairment of 5-HT descending pathway due to hyperactivity of NRM 5-HT neurons. This hyperactivity participates in mechanical pain by promoting nociceptive transmission in the spinal cord at least involving 5-HT_2A_R and 5-HT_3_R. These results suggest that therapeutic approaches aiming at downregulating the activity of NRM 5-HT neurons or pharmacologically blockade of spinal 5-HT_2A_R and 5-HT_3_R would be pertinent in the treatment of pain in PD.

## Data Availability

All data needed to evaluate the conclusions are available in the main text or the Supplementary Material. The data used to support the findings of this study are available from the corresponding author upon request.

## Abbreviations

DA: dopamine
DHSC: dorsal horn of the spinal cord
6-OHDA: 6-hydroxydopamine
HPLC: High performance liquid chromatography
NRM: nucleus raphe magnus
TH: tyrosine hydroxylase
WDR: wide dynamic range
